# Evaluation of smoking-specific and generic quality of life measures in current and former smokers in Germany and the United States

**DOI:** 10.1186/s12955-015-0316-3

**Published:** 2015-08-16

**Authors:** John E. Ware, Barbara Gandek, Anuradha Kulasekaran, Rick Guyer

**Affiliations:** John Ware Research Group, 10 Wheeler Court, Watertown, MA 02472 USA; Department of Quantitative Health Sciences, University of Massachusetts Medical School, 368 Plantation Street, Worcester, MA 01655 USA; British American Tobacco (Investments) Ltd., Group Research & Development, Regents Park Road, Southampton, SO15 8TL UK

**Keywords:** Smoking, Biomarkers of exposure, Health-related quality of life, Smoking-specific measures, Reliability, Validity

## Abstract

**Background:**

Health-related quality of life (QOL) surveys include generic measures that enable comparisons across conditions and measures that focus more specifically on one disease or condition. We evaluated the psychometric properties of German- and English-language versions of survey scales representing both types of measures in samples of current and former smokers.

**Methods:**

TQOLIT™v1 integrates new measures of smoking-specific symptoms and QOL impact attributed to smoking with generic SF-36 Health Survey measures. For purposes of evaluation, cross-sectional data were analyzed for two independent samples. Disease-free (otherwise healthy) adults ages 23–55 used a tablet to complete surveys in a clinical trial in Germany (125 current and 54 former smokers). Online general population surveys were completed in the US by otherwise healthy current and former smokers (*N* = 149 and 110, respectively). Evaluations included psychometric tests of assumptions underlying scale construction and scoring, score distributions, and reliability. Tests of validity included cross-sectional correlations and analyses of variance based on a conceptual framework and hypotheses for groups differing in self-reported smoking behavior (current versus former smoker, cigarettes per day (CPD)) and severity of smoking symptoms in both samples and, in the German trial only, clinical parameters of biomarkers of exposure.

**Results:**

Tests of scaling assumptions and internal consistency reliability (alpha = 0.71–0.79) of the smoking-specific measures were satisfactory, although ceiling effects attenuated correlations for former smokers in both samples. Correlational evidence supporting validity of smoking-specific symptom and impact measures included their substantial inter-correlation and higher correlations (than generic measures) with smoking behavior (favoring former over current groups) and CPD in both samples. In the German trial, both smoking-specific measures correlated significantly (p < 0.05) with all four biomarkers. QOL impact attributed to smoking correlated with the SF-36 mental but not physical summary measures in both samples.

**Conclusions:**

German- and English-language TQOLITv1 surveys have comparable and satisfactory psychometric properties. Cross-sectional tests, including correlations with four biomarkers, support the validity of the new smoking-specific measures for use in studies of otherwise healthy smokers. Smoking-specific measures consistently performed better than generic QOL measures in all tests of validity.

## Background

The past fifteen years have seen increased interest in the science of assessing tobacco harm reduction [1], particularly more recently for modified risk tobacco products (MRTPs) [2, 3]. In the United States, enactment of the Family Smoking Prevention and Tobacco Control Act in 2009 gave the Food and Drug Administration (FDA) authority to regulate tobacco products. While the FDA has issued draft guidance on the types of studies recommended to evaluate MRTPs [3], it has acknowledged the difficulties inherent in making premarket assessments of the effect that the introduction of a MRTP would have on the population. The FDA has encouraged development of innovative analytical methods to estimate the potential effects of MRTPs [3].

Health-related quality of life (QOL) measures have been the subject of recent FDA guidelines [4] and have been used for decades to evaluate the health status of smokers. Review of studies published over the past two decades confirmed the widespread use of the Medical Outcomes Study short-form measures (SF-36 and others) in cross-sectional and longitudinal studies of smoking behavior and identified some clear trends linking differences in smoking behavior to QOL [5, 6]. First, otherwise healthy smokers (who do not have smoking-related or other chronic conditions) typically scored above average in relation to the general population but scored worse than otherwise healthy former smokers. Second, differences between smoking groups were more apparent in measures of mental (emotional) health and general health perceptions (confidence in health). Additionally, secondary analysis of two publicly available US general population data sets [7] and data from a clinical study that involved healthy smokers switching to reduced-toxicant prototype cigarettes (RTPs) for a period of 4 weeks [8] showed that current smokers scored worse than former smokers for physical and mental health and wellbeing [9]. These results suggest that generic QOL measures also are likely to be useful in studying MRTPs, but does not address the question of whether smoking-specific QOL measures would be even more useful.

Although a number of validated questionnaires are available to assess QOL and have proven to be useful in clinical research [6, 10], generic QOL measures (which allow health conditions and treatments to be compared) and smoking-specific QOL measures (which may be more responsive to changes in smoking behavior) have not been integrated and standardized. Among the surveys used in smoking research are the: (a) Smoking Cessation Quality of Life Questionnaire, which measures generic constructs of particular importance in smoking research and self-control over smoking cessation [11, 12]; (b) Clinical COPD Questionnaire, which includes items specific to breathing problems rather than smoking [13]; and (c) PROMIS item banks and short forms measuring nicotine dependence, social motivation to smoke, and various expectancies of smoking (coping, emotional and sensory, health, psychosocial) [14]. These surveys do not comprehensively measure current symptoms, functional limitations, or other indicators of health-related QOL with attributions specifically to smoking.

The Tobacco Quality of Life Impact Tool (TQOLIT™v1) was designed to integrate smoking-specific and generic measures of QOL outcomes for current and former smokers, including those in the range of scores likely to be observed among smokers who do not have smoking-related or other chronic conditions (referred to hereafter as “otherwise healthy”). The underlying conceptual framework of TQOLITv1 includes new self-report measures of smoking-related symptoms and a new more comprehensive and standardized approach to measuring the QOL impact attributed specifically to smoking.

This paper presents the first empirical evaluation of the TQOLITv1 smoking-specific symptom and QOL impact measures and compares results across two independent US and German samples matched in terms of age and health characteristics. This evaluation addresses the assumptions underlying scale construction, score distributions and reliability and examines evidence of validity of the new smoking-specific measures in relation to a conceptual framework of hypothesized QOL determinants (i.e., smoking behavior and biomarkers of exposure) as well as differences in generic QOL outcomes that have been observed in comparisons of groups differing in smoking.

## Methods

### Samples

The German sample came from a clinical study comparing reduced toxicant prototype (RTP) and conventional cigarettes conducted in Hamburg in 2012 and approved by the independent ethics committee of the Ärztekammer Hamburg [15, 16]. Data from 125 current smokers (ages 23–55, minimum age is the legal smoking age in Germany plus 5 years) and 54 former smokers (ages 28–55) who had both TQOLITv1 and biomarker data were analyzed in this paper. Smokers had a history of regular smoking for at least 5 years, typically smoked 10 to 30 cigarettes per day (CPD) at study entry and could not be on smoking cessation medication, all as required by the trial protocol [16]. Former smokers had to have smoked at least 100 cigarettes in their lifetime, regularly smoked 10–30 cigarettes per day for at least 5 years, and quit smoking for at least 5 years at study entry. Data reported here were collected at baseline, prior to randomization to RTP or conventional cigarettes. All respondents were able to read German and self-administered the survey through an electronic data capture (EDC) system (CRF Health, Helsinki, Finland) on a tablet device with a one-item-at-a-time interface.

Data for the US matched sample were collected via an Internet survey administered in December 2011 for the NIH-sponsored Computerized Adaptive Assessment of Disease Impact (DICAT) project, which was approved by the New England Institutional Review Board. Respondents belonged to KnowledgePanel®, a representative sample of the US adult general population constructed using address-based sampling [17]. Those who reported smoking at least 100 cigarettes in their lifetime and who currently smoked every day or some days were classified as current smokers in accordance with CDC guidelines [18]. It should be noted that the US sample included current smokers reporting as few as one CPD which has the advantage of increasing variability among lighter smokers and improving tests of validity. Respondents who reported smoking at least 100 cigarettes in their lifetime but did not smoke currently and had quit at least 5 years ago were classified as former smokers. The US sample was matched to the German sample by restricting the age range to 23 through 55 (current smokers) and 28 through 55 (former smokers). All respondents were able to read English and self-administered the survey through an EDC system (QOLIX®, John Ware Research Group) with a one-item-at-a-time interface.

Respondents in Germany who reported clinically relevant gastrointestinal, renal, hepatic, neurologic, hematologic, endocrine, oncologic, urologic, pulmonary, immunologic, psychiatric, or cardiovascular disease, HIV or obesity were excluded from the clinical trial. Respondents in the US who reported matching conditions (from a checklist of 35 chronic health conditions) were excluded from the US sample.

### Measures

**Fig. 1:**
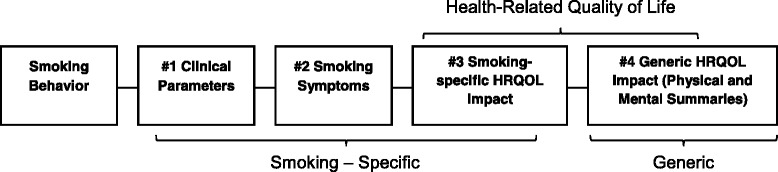
Conceptual framework for smoking-specific and generic endpoints

A conceptual framework or endpoint model is the basis for developing and evaluating evidence of validity for self-report measures of health outcomes [4]. The smoking-specific framework underlying hypotheses about results from tests of validity identifies relationships between variables (measures), along a continuum ranging from self-reported smoking behavior to the most generic health and well-being outcomes (Fig. 1). Applied to the current study of smoking behavior and QOL outcomes, this framework makes an important distinction between tests in relation to objectively-measured clinical parameters such as biomarkers of smoking exposure (box 1) and the hypothesized sequence of self-reported outcomes including smoking-specific symptoms (box 2) and the QOL impact attributed specifically to smoking (box 3). Among the hypothesized advantages of smoking-specific attributions for outcomes is greater validity than measures with attributions to health in general (box 4), in relation to both the amount and effects of smoking. An advantage of generic outcome measures is their usefulness in comparing outcomes across diseases and treatment interventions [6, 19]. We report here the first studies of whether QOL measures with attributions to smoking, as opposed to health in general, perform differently in tests of empirical validity.

The Smoking Symptoms scale was compiled by the investigators on the basis of their experience and their knowledge of the symptoms of smoking as described in the literature. It includes eight items asking about prevalent smoking-related symptoms (in order of administration): bad breath, yellowing of teeth, cold hands and feet, loss of taste and smell, nicotine stained fingers, smoker’s cough, a hoarse voice, and smell of smoke in hair and clothes. Items used a 5-choice categorical rating scale ranging from none of the time--all of the time and did not have any specific recall period. They have been extensively evaluated among current and former smokers in the US general population [9].

The Smoking Impact scale is a smoking-specific version of the QOL Disease Impact Scale (QDIS®) [9, 20], which was developed to fill the conceptual gap between disease-specific measures that do not measure quality of life and QOL measures that are not disease-specific. QDIS is the first measure to standardize the *content* and *scoring* of QOL impact attributed across specific diseases and conditions (e.g., asthma, obesity, smoking) [21]. The 49-item bank from which all QDIS forms have been constructed has been extensively evaluated using classical and modern psychometric methods, with results justifying standardization of content and scoring of an overall QOL impact scale across diseases and enabling the first norm-based scoring of disease–and condition-specific QOL impact across conditions [9]. The Smoking Impact scale administered to the German and US samples was a 7-item QDIS short-form that asked about the QOL impact of smoking on seven QOL-related content areas (in order of administration): overall quality of life, health outlook, physical functioning, fatigue, role and social functioning, and mental health (Table 1). For example, one item asked: “During the past 4 weeks, how often did your smoking limit your physical activities such as walking or climbing stairs?” with a 5-choice categorical “Never” to “Very Often” rating scale. The data reported here enabled formal tests of whether shifting items from the *generic* “health” attributions used in the SF-36® Health Survey to *smoking-specific* attributions in QDIS increased the validity of QOL impact scores in relation to other measures of smoking exposure and impact.

**Table 1:** Descriptions of smoking-specific and generic measures and biomarkers

QOL Measure^a^	Number of items	Scoring direction^b^	Worst possible score	Best possible score
Smoking-Specific				
Smoking Symptoms	8	−	Report of eight symptoms (listed in text) all of the time.	None of the 8 smoking-specific symptoms were reported.
Smoking Impact	7	−	Extreme limitations in everyday activities and quality of life attributed to smoking.	No impact on everyday activities or quality of life attributed to smoking.
SF-36 Generic				
Physical Component Summary (PCS)	35	+	Limitations in self-care, physical, social, and role activities, severe bodily pain, frequent tiredness, health rated “Poor.”	No physical limitations, pain, disabilities or decrements in well-being, high energy level, health rated “Excellent.”
Mental Component Summary (MCS)	35	+	Frequent psychological distress, social and role disability due to emotional problems.	Frequent positive affect, absence of psychological distress and limitations in usual social/role activities due to emotional problems.
Biomarker	Test	Type^c^	Constituent	Non-Tobacco Sources
4-aminobiphenyl (4-ABP)	Urine	BoE	4-ABP	Hair dyes, fried food
2-cyanoethylmercapturic acid (CEMA)	Urine	BoE	Acrylonitrile	Workplace
4-aminobiphenyl hemoglobin adduct (4-ABP-Hb)	Blood	BoED	4-ABP	Hair dyes, fried food
2-cyanoethylvaline (CEVal)	Blood	BoED	Acrylonitrile	Workplace

^a^Note: all survey items asked about the past 4 weeks except for Smoking Symptoms which asked for current status

^b^Negative (−) higher score indicates worse symptoms and quality of life; positive (+) indicates better health and quality of life

^c^BoE = Biomarker of exposure; BoED = Biomarker of effective dose

Generic outcomes were measured using the Physical (PCS) and Mental (MCS) Component Summary measures from the SF-36v2® Health Survey [7, 22]. These measures have been used in many studies of smoking [5] and have been shown to capture clinically-efficacious treatment effects in the great majority of well-controlled pharmaceutical trials across more than a dozen therapeutic areas [6].

The Smoking Symptoms and Smoking Impact scales were scored using the method of summated ratings and then were converted to norm-based scores using a linear T-score transformation to have a mean of 50 and standard deviation of 10 in the US population of ever smokers in 2011 [9]. Higher scores indicate greater frequency of symptoms and more severe quality of life impact attributed to smoking (Table 1). SF-36 summary measures were scored as recommended by their developers [22] and were normed to have means of 50 and SD = 10 in the 2011 general US population [9].

German translations of TQOLITv1 measures (Smoking Symptoms, Smoking Impact) were developed using standard methods including forward and backward translation and qualitative debriefing with lay people [23]. Translations were conducted by Mapi, Lyon, France. The International Quality of Life Assessment (IQOLA) Project German translation of the SF-36v2 was used in Germany [24].

A detailed study protocol and complete list of biomarkers for the German clinical study is published elsewhere [16], as are details regarding laboratory methods used to determine each biomarker and its accuracy [25]. Briefly, for the analyses reported here, baseline data (before any intervention) for current and former smokers were analyzed. As in a previous correlational analysis limited to smoking status, cigarettes per day (CPD) and biomarkers [25], two urinary biomarkers representing short and long term exposure, 4-aminobiphenyl (4-ABP) and 2-cyanoethylmercapturic acid, metabolites of 4-ABP and acrylonitrile respectively, which have been correlated with tobacco smoke exposure, and their related hemoglobin adducts of 4-aminobiphenyl and 2-cyanoethylvaline, which can be viewed as biomarkers of effective dose, were evaluated (Table 1).

### Analysis

This study compared the German and US TQOLITv1 in terms of data quality, tests of assumptions underlying scale construction and scoring, reliability and evidence of validity based on a conceptual framework of hypothesized relationships among smoking status and CPD, biomarkers of exposure, smoking-specific symptoms and QOL impact as well as generic QOL measures.

Tests of scaling assumptions for the new Smoking Symptoms and Smoking Impact scales included evaluation of item-total correlations corrected for item overlap and internal consistency reliability estimated with Cronbach’s coefficient alpha [26]. A minimum value of 0.30 was accepted for item-total correlations, while a reliability of 0.70 was accepted as a minimum standard for group-level comparisons [27]. In addition, descriptive statistics including the mean, standard deviation (SD) and score distributions (percent scoring at the best possible score or ceiling effect) were evaluated for new smoking-specific and SF-36 measures.

In the absence of a “gold standard” for measuring smoking-specific QOL, evidence of validity was gathered from multiple tests involving the broad framework of conceptually-related variables with which correlations would be expected for valid QOL measures, as shown in the schematic in Fig. 1 and defined in Table 1. On the strength of prior analyses correlating smoking status with biomarkers and also linking CPD to biomarkers [25], these variables were included among the tests of validity reported here. The first tests compared all measures by estimating their point-biserial correlations with smoking status (0 = former, 1 = current smoker), which is statistically equivalent to comparing group means for current and former smokers. Directional hypotheses tested included *positive* associations between smoking behavior (i.e., current status, CPD) and each of four biomarkers of exposure (Fig. 1, Box 1), Smoking Symptoms (Box 2) and Smoking Impact (Box 3); and *negative* associations between smoking and generic SF-36 measures of physical and mental impact. Further, we hypothesized that smoking behavior and the four biomarkers studied would be more strongly correlated with Smoking Symptoms and Smoking Impact measures in comparison with generic SF-36 measures. Correlations associated with chance probabilities of p <0.05 were considered significant.

For tests of the new smoking-specific and generic measures, current smokers were also divided into three groups differing in smoking-specific symptom severity: (1) None (total symptom score averaging less than “A little of the time”); (2) A little (total score averaging at least “A little of the time” but below “Some of the time”); and (3) Some (total score averaging “Some of the time” or higher). Group means were compared using one-way analysis of variance (ANOVA). The performance of the Smoking Impact and generic SF-36 scales was compared using relative validity (RV) coefficients (F-statistic for each comparator divided by the F-statistic for the most valid scale within a test). RV estimates were compared with consideration of confidence intervals estimated using empirical bootstrap [28]. As stated above, the hypothesis tested was that the smoking-specific measures would discriminate better across symptom severity groups, in comparison with the generic SF-36 measures.

Analyses were conducted in SPSS Version 19 [29] and Stata 11 [30].

## Results

The US sample of 259 ever (current and former) smokers had the same age range (23–55) as the German sample of 179 ever smokers by design and did not differ significantly in mean age (p > .05) (Table 2). A higher proportion of the US sample were male (55.2 % versus 48.6 %, respectively), although this difference was not significant at a p < .05 level. Within both samples, current smokers were younger than former smokers (p < .05), reflecting the different minimum ages (23 versus 28) in the smoking groups. The US sample had a higher percentage with some post high school education (60.6 % vs 49.2 %, p < .05). In the US population sample, current smokers also were less educated than former smokers (p < .05). Gender and race did not differ between current and former smokers in either sample (p > .05).

**Table 2:** Demographic characteristics of German and US samples

	German sample			US sample		
	Current smokers (*N* = 125)	Former smokers (*N* = 54)	Ever smokers (*N* = 179)	Current smokers (*N* = 149)	Former smokers (*N* = 110)	Ever smokers (*N* = 259)
Mean age (SD)	39.0 (9.2)	43.1 (7.8)	40.2 (8.9)	40.2 (9.8)	43.6 (8.2)	41.6 (9.3)
Range	23–55	29–55	23–55	23–55	28–55	23–55
Male (%)	48.8	48.2	48.6	55.0	55.5	55.2
Race (%)						
White	88.8	92.6	89.9	85.7	88.2	86.8
Black	0.0	0.0	0.0	7.5	6.4	7.0
Other	11.2	7.4	10.1	6.8	5.4	6.2
Education (%)^a^						
Up to high school graduate	53.6	44.4	50.8	46.3	30.0	39.4
Some post high school	24.0	22.2	23.5	37.6	27.3	33.2
College graduate or professional training	22.4	33.4	25.7	16.1	42.7	27.4

^a^German and US Ever Smoker groups differed (p < 0.05)

Item-total correlations for the Smoking Symptoms and Smoking Impact scales were substantial (r > 0.40) with few exceptions, as required for summated rating scales. Median correlations were slightly higher in the US sample than the German sample (0.49 and 0.58 vs 0.42 and 0.44 for Smoking Symptoms and Smoking Impact, respectively) (Table 3). As expected for more heterogeneous symptoms, item-total correlations for the Smoking Symptoms scale were lower than for the Smoking Impact scale. The lowest item-total correlation (0.12) was observed for the first symptom item (bad breath) in the German sample; however all others exceeded 0.30 which is satisfactory for corrected item-total correlations in a newly developed scale. While scale internal-consistency reliability was somewhat higher in the US sample, it exceeded the recommended standard of 0.70 for group comparisons for both measures in both samples.

**Table 3:** Psychometric properties of smoking-specific measures, German and US current and former smokers

	Item-Total correlations		Reliability^a^
	Median	Range	
German Sample (*N* = 179)			
Smoking Symptoms	0.42	0.12–0.58	0.71
Smoking Impact	0.44	0.33–0.62	0.72
US Sample (*N* = 259)			
Smoking Symptoms	0.49	0.37–0.62	0.76
Smoking Impact	0.58	0.48–0.72	0.79

^a^Internal consistency reliability computed using Cronbach’s alpha

Means for Smoking Symptoms and Smoking Impact measures shown to be worse for current than for former smokers in both German and US samples are documented along with other results in Table 4. For the Smoking Symptoms measure, percentages with the best possible scores (ceiling) were very low for both the US and German current smokers (4.0 % and 0.0 %) but were slightly higher for former smokers (10.9 % and 5.6 %). Similarly, the percent scoring at the ceiling for Smoking Impact was substantially lower for US and German current smokers (26.8 % and 40.8 %) in comparison with former smokers (92.7 % and 94.4 %). The skewness of the Smoking Impact measure, particularly in the German trial where 40.8 % of current smokers had the best possible score, may have constrained the correlational tests reported in Table 5. In contrast, there were no noteworthy ceiling effects (% with best possible score) for either generic measure in either sample.

**Table 4:** Means and ceiling effects of measures by smoking group and country

	German sample						US sample					
	Current smokers (*N* = 125)		Former smokers (*N* = 54)		Ever smokers (*N* = 179)		Current smokers (*N* = 149)		Former smokers (*N* = 110)		Ever smokers (*N* = 259)	
Measure^a^	Mean (SD)	% Best	Mean (SD)	% Best	Mean (SD)	% Best	Mean (SD)	% Best	Mean (SD)	% Best	Mean (SD)	% Best
Smoking-Specific												
Symptoms (−)	58.2 (8.6)	0.0	48.8 (6.6)	5.6	55.4 (9.1)	1.7	55.1(10.4)	4.0	45.3(5.4)	10.9	50.9 (9.9)	6.9
Impact (−)	50.3 (6.4)	40.8	45.6 (3.0)	94.4	48.9 (6.0)	57.0	54.0 (9.8)	26.8	45.8 (3.3)	92.7	50.5 (8.8)	54.8
SF-36 Generic												
PCS (+)	57.1 (4.3)	--	56.5 (4.2)	--	56.9 (4.3)	--	52.5 (7.6)	--	55.0 (7.1)	--	53.6 (7.5)	--
MCS (+)	54.6 (5.6)	--	53.3 (5.8)	--	54.2 (5.7)	--	47.9 (11.6)	--	50.0 (9.8)	--	48.8 (10.9)	--

^a^Negative (−) higher score indicates worse symptoms and quality of life; positive (+) indicates better health and quality of life

**Table 5:** Correlations among measures, German and US current and former smokers

Sample and measure	Scoring^a^	Current smoking status^b^	CPD	Smoking-specific symptoms	Smoking impact	PCS	MCS
German Sample (*N* = 179)							
Biomarkers							
4-ABP		0.720**	0.817**	0.439**	0.171*	0.013	0.099
CEMA		0.681**	0.773**	0.375**	0.179*	−0.006	0.064
4-ABP-Hb		0.712**	0.826**	0.538**	0.189*	−0.062	0.137
CEVal		0.659**	0.793**	0.500**	0.221**	−0.054	−0.046
Survey Measures							
Smoking Symptoms	−	0.476**	0.537**				
Smoking Impact	−	0.354**	0.251**	0.372**			
SF-36 PCS	+	0.060	0.005	−0.091	−0.099		
SF-36 MCS	+	0.104	0.107	−0.154*	−0.298**	−0.041	
US Sample (*N* = 259)							
Survey Measures							
Smoking Symptoms	−	0.491**	0.527**				
Smoking Impact	−	0.465**	0.364**	0.550**			
SF-36 PCS	+	−0.163**	−0.207**	−0.162**	−0.097		
SF-36 MCS	+	−0.098	−0.154*	−0.418**	−0.371**	-0.068	

*p < .05, **p < .01

^a^Negative (−) indicates higher score equals worse health; positive (+) indicates higher score equals better health

^b^1 = current smoker; 0 = former smoker

Note: 4-ABP = 4-aminobiphenyl; CEMA = 2-cyanoethylmercapturic acid; 4-ABP-Hb = 4-aminobiphenyl hemoglobin adduct; CEVal = 2-cyanoethylvaline hemoglobin

For comparison purposes, correlation estimates between smoking status (current = 1, former = 0) are presented in the first data column of Table 5. In further support of the conceptual framework underlying the correlational tests of validity possible in the German trial sample, significant (p < 0.05) correlations in the hypothesized direction were observed for CPD and all four biomarkers in relation to both smoking-specific measures (Table 5). Correlations with biomarker data were consistently higher for Smoking Symptoms (0.38–0.54) in comparison with Smoking Impact (0.17–0.22). In contrast, the generic physical and mental SF-36 measures did not correlate significantly with smoking status, CPD or any of the four biomarkers of exposure in the German sample. This pattern of results was replicated for the correlation of smoking-specific measures in relation to CPD in the US sample (r = 0.36–0.53, p < .01) (Table 5). (By design, biomarkers were not available for US sample respondents). Three of the four correlations with smoking status and CPD were significant (p < 0.05) for the generic physical and mental SF-36 measures in the US sample, but the magnitude of the correlations was much lower for the generic measures than the smoking-specific measures (Table 5). Smoking Symptoms and Smoking Impact scales were moderately (0.37–0.55, p < .01) inter-correlated, as hypothesized, in both samples. Correlations between smoking-specific and generic measures were higher and significant for the mental (MCS) measure (−0.15 to −0.42, p < .05) in comparison with the physical (PCS) (−0.09 to −0.16, 3 of 4 NS) measure.

Finally, evidence from tests of discrimination across groups differing in the frequency of smoking symptoms supported the hypothesized greater discriminant validity of the Smoking Impact scale over the two generic measures, in both samples (Table 6). Relative validity was consistently greatest for Smoking Impact (RV = 1.0) in both samples in comparison with the two generic measures (RV = 0.06–0.35).

**Table 6:** Relative validity (RV) of measures in discriminating among smoking symptom groups, German and U.S current and former smokers

Sample and measures		Smoking symptom frequency			F-ratio	RV
	Scoring^a^	None^b^	A little^c^	Some^d^		
German Sample		(*n* = 91)	(*n* = 79)	(*n* = 9)		
Smoking Impact	−	47.0 (4.4)	50.2 (5.8)	56.1 (12.3)	14.69**	1.00
SF-36 PCS	+	57.4 (4.4)	56.4 (4.2)	56.7 (4.4)	1.27	0.09
SF-36 MCS	+	54.4 (5.6)	54.4 (5.0)	50.0 (10.3)	2.60	0.18
US Sample		(*n* = 177)	(*n* = 74)	(*n* = 8)		
Smoking Impact	−	47.6 (5.7)	56.4 (10.7)	61.0 (10.4)	43.26*	1.00
SF-36 PCS	+	54.3 (7.7)	52.0 (6.9)	52.9 (6.7)	2.43	0.06
SF-36 MCS	+	50.7 (9.5)	46.0 (11.7)	32.7 (14.7)	15.18*	0.35

*p < 0.05

^a^Negative (−) indicates higher score equals worse health; positive (+) indicates higher score equals better health

^b^Total score less than “A little of the time” on the symptom rating scale

^c^Total score between “A little of the time” and “Some of the time” on the symptom rating scale

^d^Total score at or above “Some of the time” on the symptom rating scale

## Discussion

Overall, study results indicate that the new smoking-specific TQOLITv1 scales are likely to be useful in filling what would otherwise be conceptual and measurement gaps between smoking behaviors and symptoms and generic QOL outcomes. The Smoking Impact score estimated from responses to standardized questions differing primarily from SF-36 in terms of specific attribution to smoking was consistently more valid than the generic SF-36, in both German and US samples. Thus, along with Smoking Symptoms, the new Smoking Impact scale may advance understanding of how differences in smoking behavior and resulting differences in exposure might lead to differences in generic QOL outcomes. It is encouraging that the pattern of results from tests of psychometric properties and evidence from empirical tests of validity that could be performed for both samples was largely comparable. Conceptual and methodological issues and noteworthy limitations of the study are discussed below.

Evaluation of the conceptual framework guided by the endpoint model (Fig. 1) yielded broad evidence supporting the validity of the new Smoking Symptoms and Smoking Impact measures in relation to each other as well as hypothesized antecedents including smoking status, CPD among smokers, and objective biomarkers of exposure. The new scales were also correlated with SF-36 physical and mental measures of hypothesized generic outcomes. It is a noteworthy limitation that the evidence of validity not based on self-report was available for only the German sample. However, this is the first study that we are aware of linking independent laboratory measures of biomarkers of smoking exposure to QOL survey measures. Correlations for both Smoking Symptoms and Smoking Impact were significant with all four biomarkers in the hypothesized direction (r = 0.17–0.54) and three of eight were substantial in magnitude. The latter results supporting the validity of smoking-specific measures are in contrast to the insignificant correlations between the two generic SF-36 measures and CPD and all four biomarkers (r = 0.01–0.14, median = .05, p > 0.05) in the German trial. In contrast to the German trial, both generic measures correlated significantly with CPD in the US sample. Because very light current smokers (1–9 CPD) excluded from the German trial were included in the US sample, differences in CPD variability may be a factor underlying differences in results.

Analyses of differences in smoking behavior and smoking-specific symptoms reported here and changes in smoking behavior reported in other studies [31] suggest that there may be concurrent QOL benefits from reduced smoking exposure. In the current relatively healthy samples, the QOL benefits associated with reduced smoking exposure were particularly mental health benefits, confirming results from some previous studies [32]. Although US study participants were intentionally matched with those in the German trial in terms of age and absence of most chronic conditions, important differences in participant characteristics remained. The German sample was lower in educational level and included a higher proportion of female smokers in comparison with the US general population. These factors should be considered in generalizing study findings and could have contributed to differences in results across samples.

Translation of one of the symptom items (“bad breath”) should be evaluated further in light of the low item-total correlation for the German translation. Is this a problem of translation or cultural adaption or simply due to the heterogeneity of smoking symptoms? This important issue should be addressed using qualitative methods, which can also be applied to new items measuring smoking impact. The content of the Smoking Impact items is very similar to that of generic instruments for which qualitative evaluations have been favorable [33–35]. Whether changing the attribution of a QOL impact survey item requires additional qualitative evaluation is a matter of debate [36]. The latter was not addressed in this study and this limitation should be considered in interpreting results and addressed in future studies because it is possible that the empirical performance of generic and smoking-specific survey items could be improved on the basis of smoker-specific qualitative research.

Although the psychometric properties and performance of TQOLITv1 measures in most tests was satisfactory, skewness in score distributions (e.g., ceiling effects) was substantial particularly for smoking-specific measures among former smokers, as would be expected for relatively young smokers who are free of smoking-related chronic conditions [9]. One practical implication of ceiling effects is that estimates of the QOL benefits of quitting smoking may be attenuated. Regardless, samples of only relatively young and otherwise well adults is a shortcoming that should be noted. Although this focus on relatively young and well smokers was intended, it limits the external validity (generalizability) of findings. Future analyses also should examine the test-retest reliability of the Smoking Symptoms and Smoking Impact measures, which was not possible with the data available for this study.

Group mean scores well above the general population average were also observed for the two generic measures studied. In comparison with 2011 US population norms, average scores for relatively healthy current smokers in Germany and the US were high (close to the 70th percentile) for the SF-36 physical component summary and even higher for the mental component summary. Further study is needed to evaluate the extent to which such high scores limit the ability of generic SF-36 measures to detect QOL improvements in longitudinal studies. New generic TQOLITv1 scales designed to increase the range of reliable measurement and raise score ceilings for generic QOL measures were recently evaluated favorably [37]. Longitudinal analyses of data quality and the stability of repeated smoking-specific and generic measures are underway to evaluate their usefulness in repeated-measures outcome studies.

From the magnitude of estimates of QOL differences observed between current and former smokers and between groups differing in the severity of smoking symptoms, it is likely that QOL changes from smoking to non-smoking will be in the range typically considered an important effect size using accepted QOL standards. For example, the magnitude of generic QOL differences between current and former smokers observed in these studies is in the ballpark for minimally important differences in published comparisons from well-controlled pharmaceutical trials [6]. They also are in the range recommended as a standard for determining importance by the developers of the SF-36 [38].

## Conclusions

Despite the study limitations noted above, overall the TQOLITv1 German- and English-language surveys both enabled efficient self-administration and standardized scoring. They have comparable and satisfactory psychometric properties and sufficient empirical validity for use in German and US studies of smoking-related QOL outcomes for healthy smokers. New TQOLITv1 smoking-specific measures were consistently more valid than widely-used generic SF-36 measures across all tests for both samples. TQOLIT warrants further testing in studies evaluating changes in smoking behaviors which appear likely to be associated with noteworthy QOL outcomes.
